# Morphological Evolution and Extinction of Eodiscids and Agnostoid Arthropods

**DOI:** 10.3390/life15010038

**Published:** 2024-12-31

**Authors:** Huarui Li, Tao Dai, Yanlong Chen, Chunling Xue, Luke C. Strotz

**Affiliations:** 1 State Key Laboratory of Continental Dynamics, Shaanxi Key Laboratory of Early Life and Environments, Department of Geology, Northwest University, Xi’an 710069, China; lihuarui@stumail.nwu.edu.cn (H.L.); xuechl21@stumail.nwu.edu.cn (C.X.); lukestrotz@nwu.edu.cn (L.C.S.); 2 College of Chemical and Environmental Engineering, Hanjiang Normal University, Shiyan 442000, China; daitao10@126.com

**Keywords:** agnostids, Paleozoic, morphospace occupation, morphological evolution, extinction mechanism

## Abstract

The temporal range of eodiscids and agnostoid arthropods overlaps with several early Paleozoic geological events of evolutionary significance. However, the responses of agnostids to these events and how the perturbations associated with them (both abiotic and/or biotic) may have impacted agnostids remain uncertain. To address this uncertainty, we employ geometric morphometrics to reconstruct morphospace occupation for agnostids, thereby elucidating their evolutionary response to geological events during the early Paleozoic. The results indicate that maximum morphospace occupation was reached by Cambrian Series 2 and then declined soon thereafter. Subsequent reductions in agnostid morphospace occupation coincide not only with significant abiotic changes and associated extinction events, such as the Botoman–Toyonian Extinctions (BTEs), the Redlichiid–Olenellid Extinction Carbon Isotope Excursion (ROECE), the Drumian Carbon Isotope Excursion (DICE), and the Steptoean Positive Carbon Isotope Excursion event (SPICE), but also with major evolutionary episodes, such as the Great Ordovician Biodiversification Event (GOBE). These repeated and periodic declines in agnostid morphological diversity following Cambrian Series 2 suggest that the extinction of agnostids reflects the culmination of an episodic reduction in morphological occupancy for agnostids rather than a singular, sudden event. Accordingly, it cannot be tied to a single cause, either abiotic or biotic.

## 1. Introduction

Agnostinids and eodiscinids are diverse groups of extinct arthropods that are both widespread and abundant in Cambro–Ordovician fossil faunas, playing an important role in early Phanerozoic marine ecosystems [1,2]. While their widespread abundance and relatively rapid evolution have meant that previous work on agnostinids and eodiscinids has primarily focused on their use as biostratigraphic tools, it is these features that also make them an excellent group for exploring patterns of early arthropod evolution and animal evolution more generally [3,4,5]. In recent decades, there has been much debate surrounding the ‘agnostid problem’ since the discovery of appendages in small (larval) specimens of *Agnostus pisiformis* [6]. Many researchers have questioned the monophyly of the order Agnostida (Agnostina + Eodiscina) [7,8], as well as the classification of Agnostina as trilobites [9,10]. Moysiuk and Caron supported a nektobenthic and detritivorous lifestyle for agnostinids based on well-preserved fossil evidence from the Burgess Shale and believed that agnostinids were the sister group to polymeroid trilobites [11]. However, previous phylogenetic studies have argued that Agnostina are trilobites, and they are nested within the Eodiscina [12,13]. In recent publications, many researchers also suggest that eodiscinids and agnostinids form a monophyletic group with many similar dorsal cephalic features [14]. Thus, the monophyly of agnostids remains uncertain. Additional fossil evidence of eodiscinids is required to elucidate their soft tissue characteristics and facilitate comparisons with a broader range of trilobite taxa to fully address this ‘agnostid problem’. Here, we use geometric morphometrics to explore patterns of disparity through time and morphospace occupation in eodiscids and agnostoid arthropods. We temporarily put eodiscids and agnostoid arthropods together for the morphospace analyses, as whether they are monophyletic or not, this classification scheme will not affect the morphospace occupations of each of these two groups (see  Supplementary Text).

Several early Paleozoic events of evolutionary significance overlap the temporal range of agnostids, including the early Cambrian Stage 4 Botoman–Toyonian Extinctions (BTEs) [15,16,17,18], the Redlichiid–Olenellid Extinction Carbon Isotope Excursion (ROECE) around the Cambrian Series 2–Miaolingian boundary [19], the Drumian Carbon Isotope Excursion (DICE), a negative excursion nearly coinciding with the base of the Drumian Stage [20], the Steptoean Positive Carbon Isotope Excursion event (SPICE) that began at the base of the Paibian Stage [20,21,22,23], the Great Ordovician Biodiversification Event (GOBE) [24,25,26], and the End-Ordovician Mass Extinction (EOME) (Figure S1). These events, associated with changes in climate, sea level, and available oxygen, resulted in a significant decline in agnostid diversity, as well as evolutionary setbacks for a range of other macro-biota [15,16,17,18,19,20,21,22,23,26]. Changes in agnostid taxonomic diversity are well documented [27,28], but the morphological disparity of agnostids over time remains unknown, and the responses of eodiscinids and agnostinids to both abiotic perturbations and the radiation of other marine organisms have received limited attention [13,29,30].

In this study, we use cephalic shape to explore the evolutionary history of eodiscinids and agnostinids. It has been previously demonstrated that the shape of the arthropod head correlates with multiple organismal functions, including molting [31,32,33,34,35,36,37,38,39,40,41,42], vision [43,44,45,46], feeding [6,47], and digestive features [48,49], all of which are closely associated with the trophic niche. Thus, cephalic shape stores a wealth of evolutionary information [50]. Our primary aim is to identify if and when morphological changes occurred in both eodiscinids and agnostinids, and to determine whether these changes were gradual or episodic, aligned with known early Paleozoic geological events of evolutionary significance. An additional goal is to determine whether the decline in agnostid diversity leading up to their extinction at the end of the Ordovician coincides with a decline in morphospace occupation and functional disparity, as such patterns are often typical of clade-level extinctions [51,52]. Moreover, understanding the timing of any such decline in morphospace may better elucidate what factors were responsible for the extinction of eodiscids and agnostoid arthropods.

## 2. Materials and Methods

**Figure 1:**
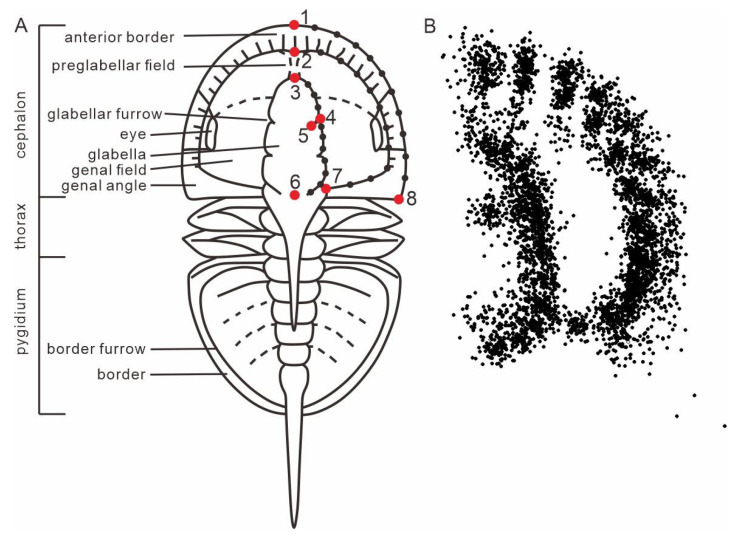
Definition (**A**) and superimposition (**B**) of landmarks (large points) and semi-landmarks (small points).

**Figure 2:**
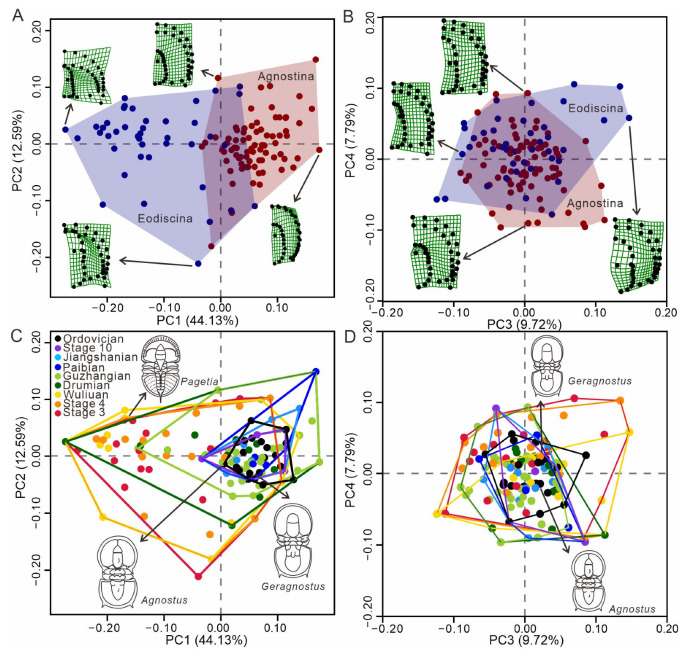
Morphospace visualization. (**A**,**B**) Morphospace occupation for the suborder Agnostina and Eodiscina with indication of characteristic thin-plate splines. All thin-plate splines represent the extreme shape of specimens for each axis. (**C**,**D**) Morphospace filling for agnostids is grouped into time bins. Sketches of individual species are indicated by arrows.

**Figure 3:**
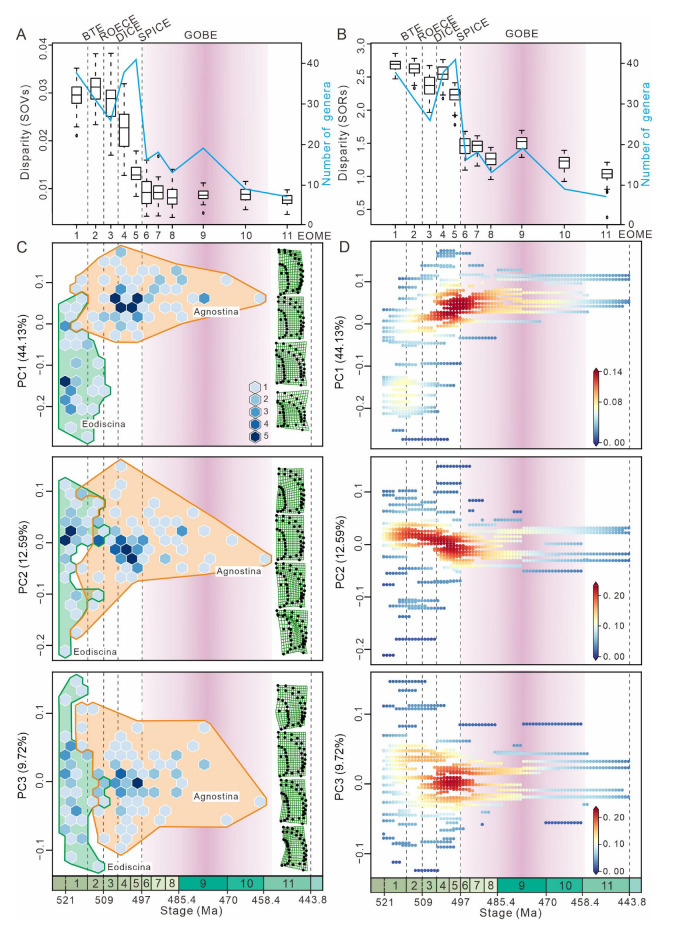
Morphological disparity and morphospace density through time. (**A**,**B**) Morphological disparity is estimated by the sum of variances (SOVs) and the sum of ranges (SORs) through time. For each time bin, the 25–75% quartiles of SORs and SOVs are shown by a box; the 95% confidence interval is shown by two horizontal lines both above and below the box; the median value of each time bin is shown by a horizontal line inside the box. (**C**) Hexagonal box plots illustrate PC1–3 values at the time of first appearance. The color scale refers to the number of genera with minimum and maximum richness of 1–5. (**D**) Scatter density plots illustrate PC1–3 values at the time of both first and last appearance. The color scale refers to density changes occupying morphospace. The density gradually increases from blue to red. BTEs—the Botoman–Toyonian Extinctions; ROECE—the Redlichiid–Olenellid Extinction Carbon Isotope Excursion; DICE—the Drumian Carbon Isotope Excursion; SPICE—the Steptoean Positive Carbon Isotope Excursion event; GOBE—the Great Ordovician Biodiversification Event; EOME—the End-Ordovician Mass Extinction; 1—Stage 3; 2—Stage 4; 3—Wuliuan; 4—Drumian; 5—Guzhangian; 6—Paibian; 7—Jiangshanian; 8—Stage 10; 9—the Lower Ordovician; 10—the Middle Ordovician; 11—the Upper Ordovician. Vertical dashed lines represent major extinction events.

A dataset for agnostids was compiled, consisting of individual fossil images for 118 genera (one image per genus) (Table S1). This represents 84% of known agnostid genera, with the remaining 16% excluded because all available images of these genera were of specimens that had undergone compression and/or deformation, or no published photos exist for that genus. The images were sourced from and screened against all formal publications related to agnostids, e.g., [1,2,53,54,55]. Several illustrations of specimens from published papers were also included in our dataset. Specimens with dorsal views, clear images and, more importantly, as little visible compression and deformation as possible, were selected preferentially. We quantified the morphology of eodiscids and agnostoid arthropods by using geometric morphometrics, focusing on their most important structure, the cephalon, which displays important sensory features [43,44,45,46] and the digestive system [48,49]. Not all agnostids possess eyes and facial sutures, so our analysis excludes these structures. As the agnostid head is symmetrical, landmarks were set only for the right side of the animal, following the method of Bault et al. [56,57] (Figure 1). For each specimen, taxonomy and age (stage) were also included in the dataset. Agnostids are included in each time bin for the analyses of morphospace as long as they range into this time bin (Figure 2C,D). Hexagonal box plots illustrate PC1–3 values for eodiscid and agnostoid arthropod genera at time of first appearance (Figure 3C). Scatter density plots illustrate PC1–3 values for agnostids genera at time of both first and last appearances (Figure 3D). We used the results to explore the morphological evolution and extinction of eodiscids and agnostoid arthropods, including the comparative relationship in morphospace occupation between the two groups, the development of morphological variation through time, and morphological disparity.

Eight landmarks and three curves (Table 1) were placed using the software TpsDig v.2.32 [58] (Figure 1). All landmarks were assigned by the first author of this study to guarantee consistency in placement. These three curves were converted into a series of 10 equally spaced semi-landmarks (Table 1). TpsUtil v. 1.78 [59] was used to generate a sliding file, which defines semi-landmarks and their sliding direction. The landmarks and sliders file were subsequently exported in the standard TPS file format (Tables S2 and S3). Generalized Procrustes Analysis (GPA) with a minimized bending algorithm was used to remove the effects of size, orientation, and position of the specimen, using TpsRelw v. 1.49 [60]. To quantify morphological variation, the variance–covariance matrix of Procrustes residual coordinates was analyzed using principal component analysis (PCA) by using PAST v3.25 [61,62]. Hexagonal box plots and scatter density plots were created using Python v. 3.11 [63] and were used to show the density of morphospace occupation through time based on the PCA.

Multiple indices were used to estimate the different aspects of disparity, such as the size, the density, and the position of the morphospace occupancy. The sum of ranges (SORs) measures the total range of morphospace occupied, while the sum of variances (SOVs) measures the average dissimilarity between points in morphospace [64]. A bootstrap analysis (999 replicates with replacement) was applied to all disparity calculations. The SOR and SOV were analyzed using R v4.2.3 [65] with the ‘dispRity’ package [66] to summarize different aspects of the differences (Table S4). Covariation and changes in the morphology of agnostids through time were tested using a nonparametric multivariate analysis of variance (NPMANOVA), also known as PERMANOVA, based on 9999 permutations carried out across all axes of variation [67]. The PERMANOVA was performed using PAST v3.25 [62].

## 3. Results

### 3.1. Geometric Morphometric Analyses

PCA for agnostid head morphology results in 72 principal components (PCs) (Table S5), with the first principal component score (PC1) representing 44.13% of the total shape variance, followed by 12.59% for PC2, 9.72% for PC3, and 7.79% for PC4 (Figure 2; Table S6).

As demonstrated by the thin-plate splines, PC1 is indicative of changes in the shape of the preglabellar area and transverse broadness, with lower values representing the absence of a preglabellar field and a long anterior border, while higher values correspond to the presence of a preglabellar field and a short anterior border. The transverse is broad at negative values and narrow at positive values. PC2 captures the shape of the glabella, with lower values representative of a glabella with a subrectangular anterior lobe and terminally angulate posterior lobe, while higher values correspond to an ogival anterior glabellar lobe and a posterior lobe that is broadly rounded posteriorly. PC3 represents changes to the transglabellar furrow (F3). Lower scores along this axis denote specimens in which F3 is weak or effaced, while higher scores denote specimens that have a well-developed F3. For PC4, this aspect of test shape variation captures the presence or absence of the anterior glabella lobe. Lower scores along this axis denote specimens in which there is an absence of an anterior lobe, while high scores along PC4 denote that the anterior lobe is elongated in a sagittal direction.

### 3.2. Morphospace Visualization and Morphological Disparity

Morphospace analyses herein illustrate the morphological variation in aspects of cephalon shape (eye shape, sutures are removed from analysis) for all agnostid taxa in our dataset, subdivided by suborder and time bins (Figure 2 and Figure S2). There is a morphological separation between each of the suborders (Figure 2A), driven largely by variation along PC1, demonstrating the considerable differences in morphology that exist between the eodiscinids and agnostinids. Most agnostinids have a long preglabellar field and a short anterior border, such as *Agnostus* and *Geragnostus*. In contrast, eodiscinids have a short preglabellar field and a long anterior border, such as *Pagetia*. Furthermore, the eodiscinids have a wider range of transverse widths (Figure 2C). These two suborders can be distinguished based on PC1, but PC2, PC3, and PC4 cannot be used to distinguish these two groups (Figure 2A,B). It is noteworthy that eodiscinids occupy a broader area of morphospace compared to agnostinids, indicating eodiscinids exhibit greater morphological variability. Eodiscids and agnostoid arthropods occupy the largest area of morphospace during the Cambrian Series 2, with total morphospace occupation decreasing in subsequent stages (Figure 2C). New areas of morphospace are colonized up until the Drumian and then from the Guzhangian onwards the total area of morphospace occupation declines. From the Paibian to the Ordovician, the total occupied morphospace declined for each time interval, increasingly confined to a smaller and smaller area of morphospace, with values increasingly concentrated in the region representing high PC1 values and close to 0 values for PC2 (Figure 2C). For PC3–4, the area of total morphospace occupation generally decreased through the Cambrian, with Ordovician taxa occupying an area of morphospace similar to that of later Cambrian forms (Figure 2D).

PERMANOVA indicates that there are significant differences in morphospace occupation for agnostids between the eleven time bins (F = 6.036, *p* = 0.0001) (Table S7). Two primary indices, the Sum of Ranges (SORs) and the Sum of Variances (SOVs), were used to examine different aspects of disparity (Figure 3A,B). The greatest amount of taxonomic diversity was during the Guzhangian, while the highest values of SOVs and SORs appeared during the Stage 4 and Stage 3, respectively. This highlights that there is a distinct decoupling between diversity and disparity. As the sum of variances is a more robust metric, the most notable disparity reduction occurred between the Drumian and the Guzhangian (Figure 3A), coinciding with reduced morphospace occupation (Figure 2C).

Hexagonal box plots (Figure 3C) show that eodiscinids reached maximum morphospace occupancy by Cambrian Stage 3, and almost no new genera appeared after either the BTE or the ROECE, and the entire clade became extinct at the end of the Guzhangian (Table S8). Conversely, agnostinids first appeared during Cambrian Stage 3 and radiated after both the BTE and ROECE (Figure 3C), occupying new areas of morphospace associated with higher PC1 values, which expand in a diamond shape. PC2 exhibits a slight shift towards higher values of PC2, while PC3 remains centered. Many new genera of agnostinids emerged after the DICE (Table S8), but none of these represent novel cephalic morphologies. Following the SPICE, morphospace occupancy declined and continued until agnostinids become extinct at the end of the Ordovician. Scatter density plots (Figure 3D) show that the majority of agnostinids occupied a relatively concentrated area of morphospace between the DICE and the SPICE. Taxonomic richness is highest in the area of morphospace represented by the dark red point (Figure 3D), with PC1 values between 0 and 0.1, and PC2 and PC3 values around 0.

## 4. Discussion

A number of previous studies have shown that maximum morphospace occupancy is reached rapidly, early in the history of a clade before subsequently declining, with later values never returning to near the early maximum [68,69,70,71]. This rapid rise and then decline could reflect the fact that ecological opportunities are greater in the early history of many clades, diminishing as ecological niches became occupied and then saturated, and the increase in disparity may also be restricted by extrinsic factors (e.g., ecological, physical), which causes evolutionary pressures [50,72,73,74,75]. Our results demonstrate that agnostids are consistent with this early-burst evolutionary model, reaching their maximum morphological diversity early in their evolutionary history (Cambrian Series 2) and declining thereafter, with almost no new morphospace occupation from the Drumian until their extinction at the end of the Ordovician (Figure 3).

It has been previously demonstrated that head morphology captures multiple organismal functions, including molting [31,32,33,34,35,36,37,38,39,40,41,42], vision [43,44,45,46], feeding [6,47], and digestion features [48,49]. However, due to limited functional morphological research linking the ecology, physiology, and head morphology of agnostids, it is difficult to explain why they did not undergo a morphological expansion after Cambrian Stage 4 (Figure 3D).

### 4.1. Response to the Cambrian Geological Events

The Eodiscina and Agnostina show different responses to the BTE and ROECE. Both the BTE and ROECE have been linked to animal extinction events [15,20] and coincide with a large reduction in morphospace occupation for eodiscinids (Figure 3C). The decline in eodiscoid morphospace occupancy in the Cambrian may be because eodiscinids are thought to be scavengers that feed at the benthos [6], and therefore the sea-level changes and anoxia associated with both the BTE and ROECE would have had a disproportionate and negatively impact on them [16,17,18,19,20,47,76], resulting in the removal of a number of distinct morphologies, such as cephala with a long anterior border but without a preglabellar field. In contrast, both the BTE and ROECE resulted in agnostinids expanding into new areas of morphospace never occupied by eodiscinids, including a longer preglabellar field and a shorter anterior border (Figure 3C). These morphological explorations, which were different between agnostinids and eodiscinids, may be related to changes in oxygen [16,18]. In addition, there were intervals where specific areas of morphospace were densely occupied by agnostinids between the DICE and the SPICE events (Figure 3D). This could be due to subjective factors in previous studies that resulted in the over-splitting of eodiscids and agnostoid arthropod taxa. Some of these taxa with similar morphologies could be synonymous taxa. DICE and SPICE are, respectively, associated with two negative and positive marine carbon isotope excursions, which are considered to represent global carbon cycle perturbations [20,21]. The SPICE event occurred at the base of the Paibian Stage, which coincided with a notable trilobite extinction event and could be related to environmental factors such as the widespread oceanic anoxia and sea-level changes [20,77]. Agnostinids were also affected, with a decrease in total morphospace occupation, which can also be observed in the SORs and SOVs (Figure 2C,D and Figure 3A,B).

For the Cambrian, our results identify that episodes in agnostid evolution were mainly influenced by abiotic factors such as anoxia and sea-level changes [16,17,18,20,47,76,77]. It is plausible, based on our results, that there are multiple declines of eodiscinid and agnostinid morphospace occupation during the Cambrian, which correspond with individual anoxic events. The BTE, ROECE, and SPICE have all been identified as anoxic events [16,20,77]. Eodiscinids is severely impacted by both the BTE and ROECE, while the SPICE reduced the morphospace occupation of agnostinids (Figure 3C,D).

### 4.2. Response to the GOBE

The Great Ordovician Biodiversification Event (GOBE) represents a large biological radiation believed to have been facilitated by pulses in atmospheric oxygen [78] as well as many other factors (for example, increasingly complex pelagic communities) [79], which served to expand the proportion of habitable ocean and provided new ecological opportunities [26]. However, neither morphological nor taxonomic diversity of agnostinids increased during the GOBE; on the contrary, there was a reduction in morphospace occupation and taxonomic diversity (Figure 3C,D). Our data suggest that the increase in diversity during the GOBE may be restricted to only part of the Ordovician biological groups. We propose several possibilities, both direct and indirect, that may explain the observed morphospace decline for agnostinids. Firstly, the temperature in the Early Ordovician was quite high, with sea-surface temperatures estimated to be as high as 45 °C [80]. In this supergreenhouse state [81], vulnerable marine organisms would have been forced to shelter in deeper (or cooler) places to survive, causing adaptive changes to temperature. The soft tissue morphology of *Peronopsis* and *Ptychagnostus* from the middle Cambrian (Wuliuan Stage) Burgess Shale suggests that agnostinids have a nektobenthic and detritivorous lifestyle [11]. The high temperatures of the Early Ordovician may have forced agnostinids into limited refugia, stagnating ecological expansion. Secondly, the species diversity of trilobites reached its peak in the Early Ordovician [82] and these new benthic trilobite taxa that first appeared during the GOBE [27,47] may have been direct competitors with agnostinids. Some of the newer trilobite groups started to expand into pelagic environments at that time [83] and new morphologies developed, suggesting the possibility of direct competition with agnostinids. Additionally, it is unlikely that the GOBE is a single event, but is composed of a series of events, each of which had varied impacts on different groups and in different regions [25]. According to a high-resolution summary of marine invertebrate biodiversity, the taxonomic diversity of trilobites generally declined during the GOBE but then fluctuated during the Middle–Late Ordovician [82,84,85]. Focusing on the diversity of agnostinids at the clade level, there are very different patterns, which is consistent with these previously observed results from the GOBE [82].

### 4.3. Extinction Mechanism

Some morphotypes have existed from the Cambrian through to the Ordovician, such as the forms represented by *Agnostus* and *Geragnostus* (Figure 2C,D). These morphotypes match (more or less) with the morphospace overlap between agnostinids and eodiscinids along PCs 1–4 (Figure 2A,B). Although the occupied area of morphospace was decreasing from the Cambrian to the Ordovician, the distribution tends to converge towards a specific area, possibly representing extinction selectivity.

These repeated and periodic declines in agnostid morphological diversity following Cambrian Series 2 directly correlate with biological competition, anoxia, sea-level changes, and high sea-surface temperature [16,17,18,20,47,77,80,81]. This increasingly restricted morphological (and associated ecological) diversity possibly served to increase the possibility of total extinction of eodiscids and agnostoid arthropods due to unforeseen contingent events, which eventually came in the form of the End-Ordovician glaciation event, resulting in the extinction of eodiscids and agnostoid arthropods along with many other groups as part of the associated mass extinction event [86].

## 5. Conclusions

(1) Our data indicate that anoxia from the BTE, ROECE, and SPICE events [16,20,77] may be the main reason for the periodic decline of the morphospace occupation of eodiscids and agnostoid arthropods, as anoxic events seemingly eliminated a large number of morphological types and reduced the overall morphospace of the clade.
(2) During the Ordovician, abiotic factors such as temperature, as well as biotic factors such as competition, may have led to a reduction in morphospace occupation for the agnostinids. There was no observable increase in agnostinids morphological disparity during the GOBE, unlike for many groups.
(3) After the Cambrian Series 2, the repeated and periodic decline in morphological disparity of eodiscids and agnostoid arthropods is consistent with multiple geological events during the Cambrian–Ordovician. The extinction of eodiscids and agnostoid arthropods is likely the result of a stepwise decrease in the total morphological occupancy, rather than a single, sudden event, and accordingly cannot be tied to a single cause, either abiotic or biotic.

## Figures and Tables

**Table 1:** Definition of landmarks and semi-landmarks. Figure 1A for visual.

Landmark Code	Description
1	Intersection between sagittal line and anterior cephalic margin
2	Intersection between sagittal line and anterior preglabellar field
3	Anterior-most point of sagittal glabellar length
4	Intersection between F3 and dorsal furrow
5	Point of the greatest extent medially of the F3
6	Posterior-most point of sagittal glabellar length
7	Posterior-most end of genal field
8	Extremity of genal angle or spine
9–18	Sliders between landmarks 1 and 8, along the margin of cephalon
19–28	Sliders between landmarks 2 and 7, along the margin of genal field
29–38	Sliders between landmarks 3 and 6, along the margin of glabella

## Data Availability

The data presented in this study are available in this article and Supplementary Materials.

## Supplementary Materials

The following supporting information can be downloaded at https://www.mdpi.com/article/10.3390/life15010038/s1, Table S1: Dataset; Table S2: Landmarks data; Table S3: Sliders file; Table S4: Data; Table S5: PCA data; Table S6: Eigenvalue data; Table S7: PERMANOVA; Table S8: The number of genera; Figure S1: The timing of various evolutionary episodes and extinction events. Figure S2: Morphospace visualization of the Ordovician; Supplementary Text.
