# Supplementary material for: Morphological Evolution and Extinction of Eodiscids and Agnostoid Arthropods
Source: Life (Basel). 2024 Dec 31;15(1):38. doi: 10.3390/life15010038 (PMC11766919; doi:10.3390/life15010038)
Supplement: Supplementary file 1 [file life-15-00038-s001.zip › S4-Data.pdf]

```
library(ape)
library("dispRity")

rt<-read.csv(file = "1.csv")
mydata <- as.matrix(rt)
mygroup <- list("1" = mydata[c(1:38)], "2"= mydata[c(39:69)],
                "3" = mydata[c(70:95)], "4"= mydata[c(96:133)],
                "5" = mydata[c(134:174)], "6"= mydata[c(175:190)],
                "7" = mydata[c(191:208)], "8"= mydata[c(209:221)],
                "9"=          mydata[c(222:240)],          "10"=mydata[c(241:249)],"11"=
mydata[c(250:256)])
rownames(mydata)<-rt[,1]
mydata
mydata<-mydata[,-c(1:4)]
head(mydata)

##sum of variances
disparity_data <- dispRity.per.group (data=mydata, group = mygroup, metric = c(sum, variances))
disparity_data
summary(disparity_data, digits=4)
plot(disparity_data, ylab="Disparity (SOVs)")

##sum of ranges
disparity_data_ranges <- dispRity.per.group(mydata, group = mygroup, metric = c(sum, ranges))
disparity_data_ranges
plot(disparity_data_ranges, ylab="Disparity (SORs)")
```

```python
import numpy as np
import matplotlib.pyplot as plt
from matplotlib import rcParams
import pandas as pd

import matplotlib.colors as colors
def truncate_colormap(cmap, minval=0.0, maxval=1.0, n=100):
	new_cmap = colors.LinearSegmentedColormap.from_list(
		"trunc({n},{a:.2f},{b:.2f})".format(n=cmap.name, a=minval, b=maxval),
		cmap(np.linspace(minval, maxval, n)),
	)
	return new_cmap
filename = r'C:\Users\Administrator\1.xlsx'
x_name = 'time'
y_name = 'PC 1'
gridsize = 18
cmap = "Blues"
cmap = plt.get_cmap(cmap)
cmap = truncate_colormap(cmap, 0.2, 1)
mincnt = 1
fig = (3.3,2)
edgecolor = 'white'
config = {"font.family":'Arial', "font.size": 6, "mathtext.fontset":'stix'}
rcParams.update(config)
df2=pd.read_excel(filename)
x=df2[x_name].values
y=df2[y_name].values
tips = df2[[x_name,y_name]]
vmax =5
tips.plot.hexbin(x=x_name, y=y_name,mincnt=mincnt, gridsize=gridsize, cmap=cmap, figsize=fig,
edgecolors=edgecolor, vmax=vmax,linewidths=0.5)

plt.ylabel("PC1")
plt.xlabel("Time(Ma)")
plt.xlim(525,440)
plt.savefig(y_name+'.svg')

plt.show()
```

```
import numpy as np
import matplotlib.pyplot as plt
import numpy as np
from scipy.stats import gaussian_kde
import pandas as pd
from matplotlib import rcParams
import matplotlib.colors as colors
def truncate_colormap(cmap, minval=0.0, maxval=1.0, n=100):
    new_cmap = colors.LinearSegmentedColormap.from_list(
        "trunc({n},{a:.2f},{b:.2f})".format(n=cmap.name, a=minval, b=maxval),
        cmap(np.linspace(minval, maxval, n)),
    )
    return new_cmap
filename=r'C:\Users\Administrator\2.xlsx'
x_name = 'time'
y_name = 'PC 1'
fig = (3.1,2)
config = {"font.family":'Arial', "font.size": 6, "mathtext.fontset":'stix'}
marker='o'
s=1
cmap='RdYlBu_r'
cmap = plt.get_cmap(cmap)
cmap = truncate_colormap(cmap, 0,1)
label='Frequency'
vmin=0
vmax=0.23
rcParams.update(config)
df2=pd.read_excel(filename)
x=df2[x_name].values
y=df2[y_name].values
xy = np.vstack([x,y])
z = gaussian_kde(xy)(xy)
idx = z.argsort()
x, y, z = x[idx], y[idx], z[idx]
fig,ax=plt.subplots(figsize=fig,dpi=100)
scatter=ax.scatter(x,y,marker=marker,c=z,s=s,label='LST',cmap=cmap,vmin=vmin, vmax=vmax)
cbar=plt.colorbar(scatter,shrink=1,orientation='vertical',extend='both',pad=0.015,aspect=30,label
=label)
plt.ylabel("PC1")
plt.xlabel("Time(Ma)")
plt.xlim(525,440)
plt.show()
```
