# Supplementary material for: Morphological Evolution and Extinction of Eodiscids and Agnostoid Arthropods
Source: Life (Basel). 2024 Dec 31;15(1):38. doi: 10.3390/life15010038 (PMC11766919; doi:10.3390/life15010038)
Supplement: Supplementary file 1 [file life-15-00038-s001.zip › Supplementary Text.pdf]

Morphological Evolution and Extinction of Eodiscids and Agnostoid Arthropods

Huarui Li [1], Tao Dai [2], Yanlong Chen [1,*], Chunling Xue [1] and Luke C. Strotz [1]

Separate analyses of eodiscids and agnostoid arthropods

Analyzing the two groups separately reveals that the maximum morphospace occupation of eodiscids in PC1–2 was reached by Cambrian Stage 3 (Figure S3A), subsequently decreasing over time, ultimately leading to the extinction of eodiscids after the Guzhangian. In PC3–4, there was morphological innovation with new taxa occupying lower PC4 values during Cambrian Stage 4, followed by a further reduction in morphospace occupancy after the Wuliuan (Figure S3B). Agnostoid arthropods initially increase and then decrease in morphospace occupation over time in PC1–2, with morphological innovation associated with taxa represented by higher PC1 values during the Wuliuan (Figure S3C). Agnostoid arthropods reach their peak in morphospace during the Drumian and then subsequently shrank over time. For PC3–4, morphological innovation is associated with taxa represented by higher PC3 values and lower PC4 values during Cambrian Stage 4 (Figure S3D). Further expansion along PC4 continued into the Wuliuan, indicating the emergence of new morphologies from Cambrian Stage 4 to the Wuliuan.

Separate analyses of the morphospace occupied by both eodiscids and agnostoid arthropods are similar to the analyses of these two groups together. The shape of the morphospace occupation of eodiscids when both eodiscids and agnostoid arthropods are assessed collectively (Figure 2) is the same as the morphospace occupation of eodiscids when analyzed alone (Figure S3A,B and S4A). This is also the case for agnostoid arthropods (Figure 2, S3C,D and S4B). This is likely because the relative distances between these specimens in the morphospace are the same whether these two groups are analyzed collectively or separately. Based upon the ‘separate analysis’, it seems that the 'early burst' in morphological change, which is proposed based upon the combined analysis, is dominated by eodiscids (Figure S4).

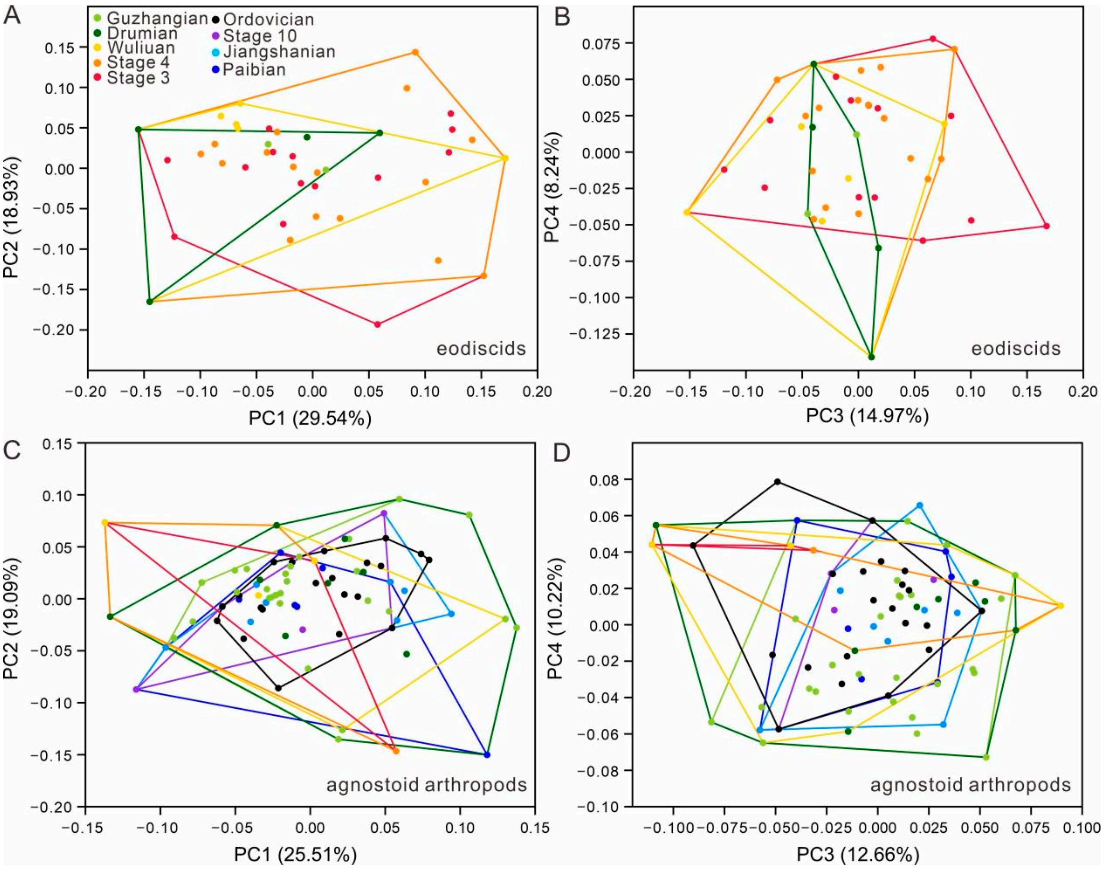

Figure S3. Morphospace visualization. (**A,B**) Morphospace filling for eodiscids is grouped into time bins. (**C,D**) Morphospace filling for agnostoid arthropods is grouped into time bins.

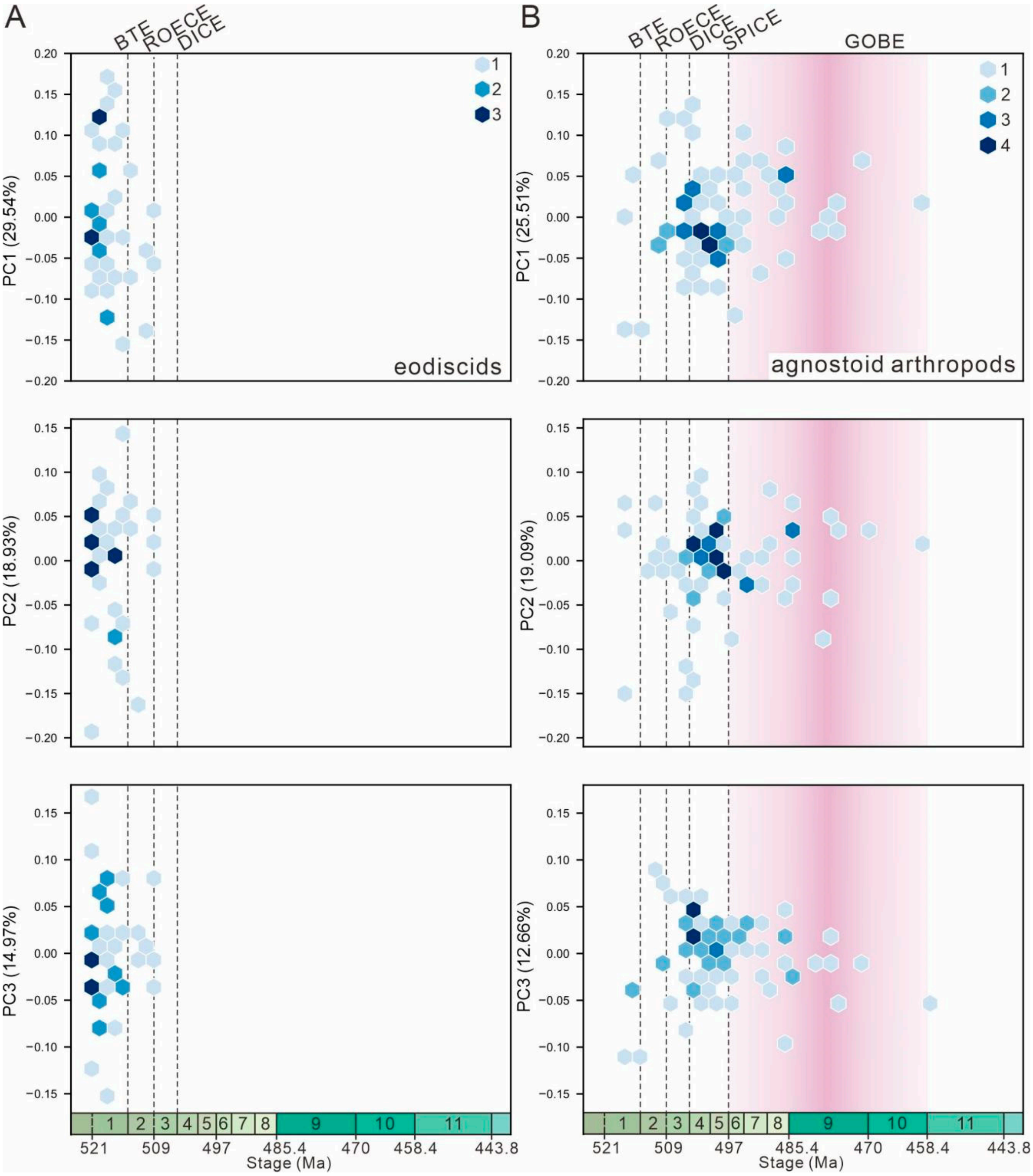

Figure S4. Morphospace density through time. (**A**) Hexagonal box plots illustrate PC1–3 values of eodiscids at the time of first appearance. (**B**) Hexagonal box plots illustrate PC1–3 values of agnostoid arthropods at the time of first appearance. The color scale refers to the number of genera with minimum and maximum richness. BTEs—the Botoman–Toyonian Extinctions; ROECE—the Redlichiid–Olenellid Extinction Carbon Isotope Excursion; DICE—the Drumian Carbon Isotope Excursion; SPICE—the Steptoean Positive Carbon Isotope Excursion event; GOBE—the Great Ordovician Biodiversification Event; EOME—the End-Ordovician Mass Extinction. 1—Stage 3; 2—Stage 4; 3—Wuliuan; 4—Drumian; 5—Guzhangian; 6—Paibian; 7—Jiangshanian; 8—Stage 10; 9—the Lower Ordovician; 10—the Middle Ordovician; 11—the Upper Ordovician. Vertical dashed lines represent major extinction events.
