# Supplementary figures and images for: Morphological Evolution and Extinction of Eodiscids and Agnostoid Arthropods
Source: Life (Basel). 2024 Dec 31;15(1):38. doi: 10.3390/life15010038 (PMC11766919; doi:10.3390/life15010038)

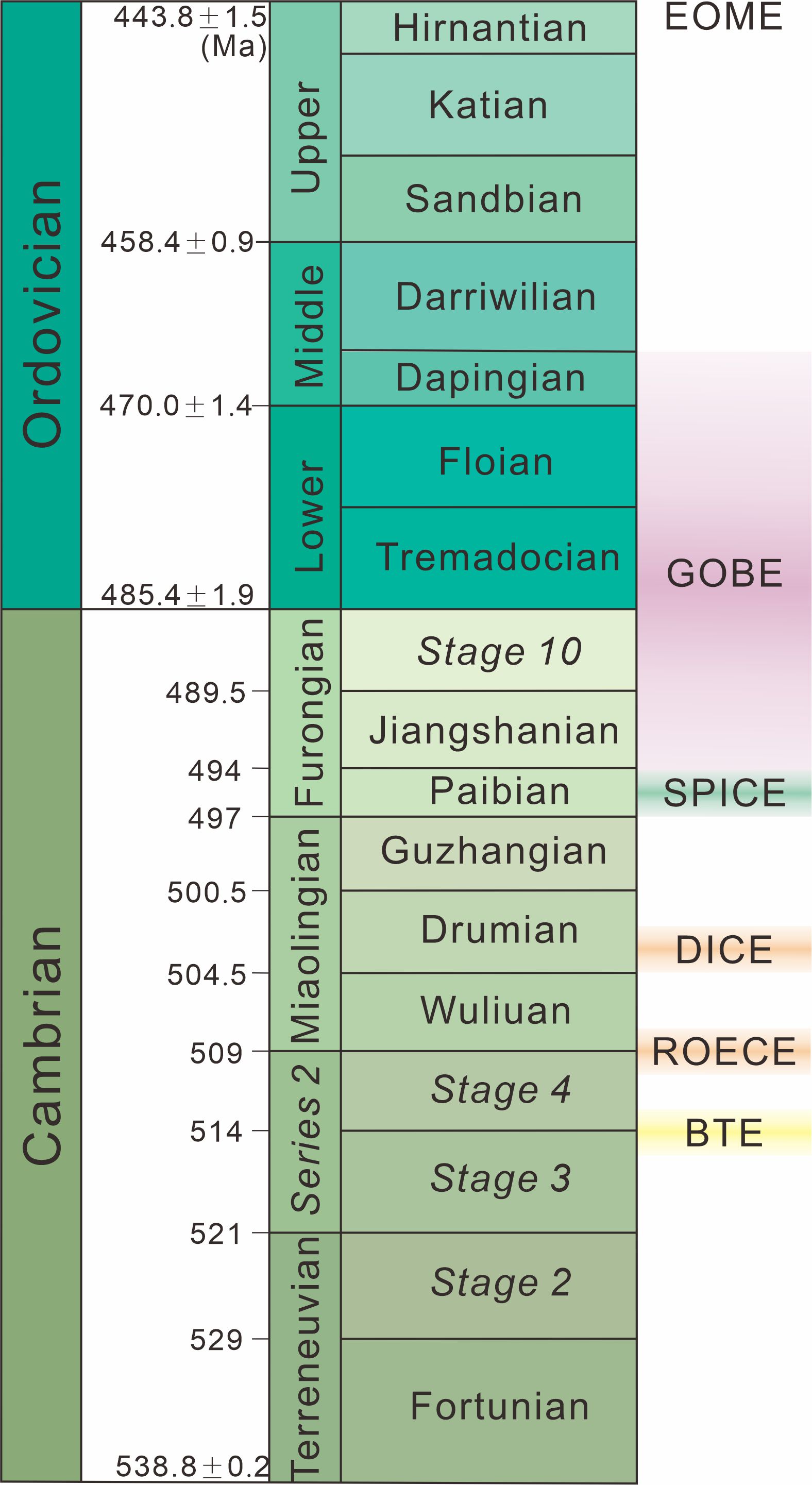

Supplement: Supplementary file 1 [file life-15-00038-s001.zip › Figure S1.jpg]

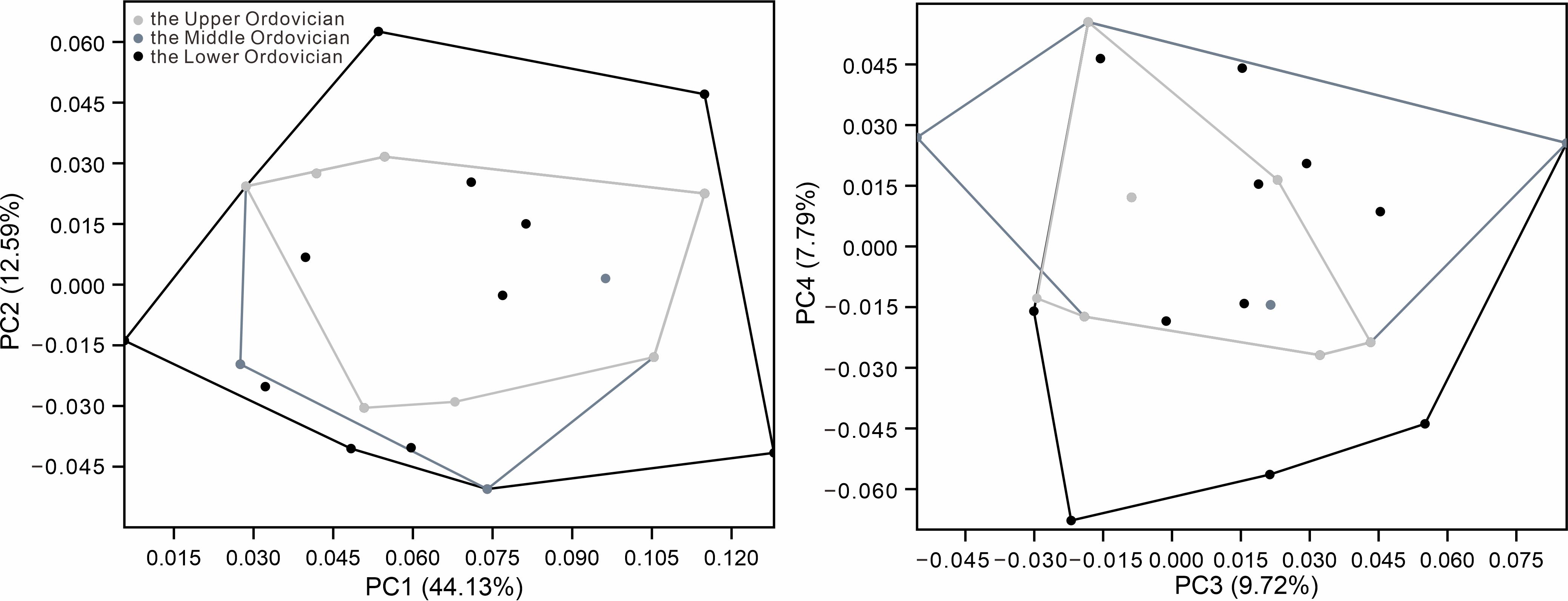

Supplement: Supplementary file 1 [file life-15-00038-s001.zip › Figure S2.jpg]
